# Early Epinephrine Improves the Stabilization of Initial Post-resuscitation Hemodynamics in Children With Non-shockable Out-of-Hospital Cardiac Arrest

**DOI:** 10.3389/fped.2019.00220

**Published:** 2019-06-06

**Authors:** Yan-Ren Lin, Chao-Jui Li, Cheng-Chieh Huang, Tsung-Han Lee, Tren-Yi Chen, Mei-Chueh Yang, Chu-Chung Chou, Chin-Fu Chang, Hsi-Wen Huang, Hsiu-Ying Hsu, Wen-Liang Chen

**Affiliations:** ^1^Department of Emergency Medicine, Changhua Christian Hospital, Changhua City, Taiwan; ^2^School of Medicine, Chung Shan Medical University, Taichung City, Taiwan; ^3^School of Medicine, Kaohsiung Medical University, Kaohsiung City, Taiwan; ^4^Department of Emergency Medicine, Kaohsiung Chang Gung Memorial Hospital, Chang Gung University College of Medicine, Kaohsiung City, Taiwan; ^5^Department of Leisure and Sports Management, Cheng Shiu University, Kaohsiung City, Taiwan; ^6^Department of Biological Science and Technology, National Chiao Tung University, Hsinchu, Taiwan; ^7^Department of Nursing, Dayeh University, Changhua City, Taiwan; ^8^Department of Nursing, Changhua Christian Hospital, Changhua City, Taiwan

**Keywords:** epinephrine, children, OHCA, survival, non-shockable, time, early, outcome

## Abstract

**Background:** In children with non-shockable out-of-hospital cardiac arrest, early epinephrine (EE) might help to establish the return of spontaneous circulation (ROSC) and be associated with survival. In the present study, we aimed to analyze the effects of EE on outcomes and post-resuscitation hemodynamics in children with non-shockable OHCA.

**Methods:** This was a retrospective analysis of data from 216 children (<19 years) who had suffered non-traumatic and non-shockable OHCA and received epinephrine for resuscitation (Jan 1, 2006–Dec 31, 2014). Demographics, pre-/in-hospital information, and the time to the first dose of epinephrine were recorded. Early post-resuscitation hemodynamics (the first hour after sustained ROSC), survival and good neurological outcomes (Pediatric Cerebral Performance Category Scales 1 or 2) were analyzed by the time to epinephrine—classified as early (EE): <15 min, intermediate (IE): 15–30 min, or late (LE): >30 min.

**Results:** Twenty-eight (13.0%) children survived to discharge, but only 17 (7.9%) had good neurological outcomes. In all, 41 (18.9%) children received EE; in comparison to IE and LE, this was significantly associated with tachycardia (73.9%) in the post-resuscitation period (*p* < 0.05). Tachycardia (OR: 7.41, 95% CI: 1.96–29.31) and hypertension (OR: 6.03, 95% CI: 1.85–13.77) were significantly associated with EE after adjusting for confounding factors. EE was also significantly associated with better overall outcomes than ME and LE (any ROSC, sustained ROSC, survival to the intensive care unit, admission, survival to discharge and good neurological outcomes, all *p* < 0.05).

**Conclusions:** EE helped to establish ROSC but was also associated with more tachycardia and hypertension in the early post-resuscitation period. In children with non-traumatic and non-shockable OHCA, EE was associated with a higher survival rate and better neurological outcomes than were ME and LE.

## Introduction

The outcomes of children who suffer from non-traumatic out-of-hospital cardiac arrest (OHCA) are very poor (1–3). Only 1 to 19.6% of survivors have been reported as exhibiting good neurological outcomes (4–7). Several factors that could improve the chance of survival have been identified, including bystander cardiopulmonary resuscitation (CPR), initial shockable rhythm, *chest compression-only CPR* and shorter duration of transportation (3, 5, 7–12). Early epinephrine (EE) administration might be associated with survival, but may also exhibit both beneficial and harmful effects, as has been generally discussed in adult OHCA patients (13–15). Some studies have indicated that EE might help to establish spontaneous circulation during CPR by elevating cardiac output (β-agonist effects, increased heart rate, and contractility). The onset of action appears to be very rapid, with a half-life of only 5–10 min (16–18). However, other studies have suggested that the effect of EE may be limited. It also exhibits an intense α-agonist effect (the non-selective effect of epinephrine), leading to early vasoconstriction, increased tissue oxygen consumption and *poor end-organ perfusion* (19–23). Some studies have even reported that the harmful effects would be more obvious in end organs [i.e., limbs, brain, intestine, kidney; (19, 24, 25)]. More importantly, these effects would potentially be associated with poor neurological outcomes (21).

Thus, the controversial effects of EE raise several issues for emergency department (ED) physicians treating OHCA patients. Therefore, some large population studies have analyzed the patients' outcomes with different times to epinephrine administration. The overall effects of EE seem to be associated with better survival and neurological outcomes in adult OHCA patients (13, 26, 27). However, in OHCA children, information on the beneficial or harmful effects of EE is still limited. Although a few studies have reported that EE might increase the chance of achieving sustained return of spontaneous circulation (ROSC) and even survival in children with in-hospital cardiac arrest (IHCA), the detailed mechanism of the restoration of spontaneous circulation in early post-resuscitation periods is not clear (28, 29). Furthermore, the detailed effect of EE on post-resuscitation hemodynamics has not been well-addressed in non-traumatic OHCA in children.

It has been demonstrated that early defibrillation can establish spontaneous circulation more effectively than EE in patients with shockable rhythm [ventricular fibrillation (VF) and pulseless ventricular tachycardia (VT)] (13, 30). Therefore, the present study focusses on the effect of EE on non-shockable rhythms [asystole and pulseless electrical activity (PEA)]. We aim to analyze the effects of EE treatment on post-resuscitation hemodynamics in children with non-shockable and non-traumatic OHCA.

## Materials and Methods

### Study Design

The data of this study were retrospectively collected from the two medical centers—one in central and one in southern Taiwan (January 1, 2006 to December 31, 2014). Children (<19 years) were included who had suffered non-traumatic OHCA and received epinephrine for resuscitation. Patients' outcomes and post-resuscitation hemodynamics were analyzed according to the time to epinephrine treatment (early: <15 min, intermediate 15–30 min, late >30 min). The cut-off time points in this study were determined in accordance with a review of the literature (31–33).

### Ethics Statement

This study was carried out in accordance with the recommendations of the STROBE statement. The protocol was approved by the institutional review board (IRB) of Changhua Christian Hospital (central Taiwan). Since this is a retrospective chart review study, and outputs were secondarily anonymized data, informed consent was not needed under the IRB permission. Finally, the team members and data collectors also followed the rules of the IRB.

1. ***Full name of the ethics committee:*** Institutional review board (IRB) of Changhua Christian Hospital.
2. ***Consent procedure:*** retrospective chart review; no formal consent necessary.
3. ***Any additional considerations of the study in cases where vulnerable populations were involved, for example minors, persons with disabilities or endangered animal species:*** There were no additional considerations for vulnerable populations under the IRB permission.

### Inclusion and Exclusion Criteria

#### Inclusion

Children (<19 years) who had suffered non-traumatic OHCA and received epinephrine for CPR.

#### Exclusion

1. Cardiac arrest caused by trauma, intoxication, drowning, child abuse, or substance abuse.
2. Absence of epinephrine treatment—either prehospital or ED or during CPR. Patients with a “Do Not Resuscitate” order were also excluded.
3. Unknown time of collapse or time to first epinephrine.
4. Incomplete medical records
5. Initial shockable cardiac rhythm (VF/pulseless VT).

### Study Setting and Population

Information on demographics and resuscitation was retrospectively obtained from the medical records (and CPR database). Prehospital information, including duration of prehospital CPR, duration from time of collapse to arrival of emergency medical services (EMS), time from EMS arrival to epinephrine, time from the scene of collapse to hospital, and witness of the arrest were obtained from EMS records or witnesses (i.e., EMS personal, family member, caregiver). The ages of children were classified into groups (infant: <1, toddler: 1–4, preschool children: 5–9, schoolchildren: 10–14, and adolescents: 15–19 years). The possible etiologies of arrest were divided into the following seven major causes: sudden infant death syndrome, cardiovascular diseases, neurological diseases, respiratory diseases, malignancy, infections, and others (including idiopathic causes). The ED resuscitation information included the duration of in-hospital CPR and initial cardiac rhythms. The rhythms included asystole and PEA and were obtained from an electrocardiography (ECG) monitor (on arrival at the ED).

### Time to Epinephrine

Information on epinephrine administration was also obtained from the medical and EMS records. The route of the first epinephrine administration was given as intravenous or non-intravenous. Time to epinephrine was defined as the duration from collapse (no pulse, no vital signs, and/or unconsciousness) to the first dose of epinephrine. The time of collapse was decided according to EMS records, witness statements and/or medical records. As given in the exclusion criteria, children were excluded if the time of collapse could not be confirmed. Time to epinephrine was classified as early (<15 min), intermediate (15–30 min), or late (>30 min).

### Study Protocol

#### Primary Outcomes—Utstein Style Reports for All Patients

All patients' outcomes were classified using Utstein style reports [any ROSC, sustained ROSC, survival to admission to intensive care unit (ICU), survival to discharge, good neurological outcomes] (34, 35). The “good neurological outcomes” in this study were considered as Pediatric Cerebral Performance Category Scales (PCPCS) = 1 or 2 on discharge from hospital (36, 37). Outcomes were classified by time to epinephrine.

#### Secondary Outcomes—Post-resuscitation Hemodynamics (in the First Hour After Achieving Sustained ROSC) in Patients who Survived to ICU Admission

The secondary outcomes were analyzed by time to first treatment with epinephrine.

#### Post-resuscitation Hemodynamics

Post-resuscitation hemodynamics were recorded in the first hour after achieving sustained ROSC (10, 11). The variables included oxygenation (hypoxia and non-hypoxia), heart rate (tachycardia, normal, bradycardia), heart rhythm (sinus, non-sinus), mean blood pressure (hypertension, normal, hypotension), consciousness level (Glasgow Coma Score, GCS>7, 4–7, = 3), body temperature (pyrexia and non-pyrexia), and urine output (>1, 1–0.5, <0.5 ml/kg/h). In addition, oxygenation was evaluated with ventilator support. The verbal response in patients who received endotracheal intubation was classified as a single score. Pyrexia was defined as over 38 degrees Celsius (°C). The post-resuscitation care protocols in ED or ICU adhered to the recommendations of the American Heart Association (AHA) guidelines during the study period. The protocols included systemic supports (respiratory, cardiovascular, neurological systems), continuous monitoring (blood pressure, cardiac rhythms, and oxygenation), end organ protection (brain and kidney), and aggressive fever control. Temperature was continuously measured. Temperatures of 38°C or more were aggressively treated in all pediatric patients after ROSC (38–40).

### Data Analysis

SPSS software version 15.0 (SPSS Inc., Chicago, IL) was used to analyze the data in this study. Descriptive statistics for demographics, pre-/in-hospital resuscitation information, hemodynamics and primary/secondary outcomes are reported as the number, percentage and/or mean ± standard deviation (SD). The relationships between different times to epinephrine and post-resuscitation hemodynamics were analyzed with the chi square test. Multinomial logistic regression analysis was used to determine the effect of EE, taking LE as the reference group, for hemodynamics parameters, pre/in-hospital CPR information, body temperature, and etiology of arrest. Finally, the primary outcomes of patients were also analyzed by the chi square test by different times to epinephrine. *p* < 0.05 was considered statistically significant.

## results

### Demographics of Non-traumatic OHCA Children

The final population of this study was 216 children. Demographics and resuscitation information are shown in Table 1. Most patients were infants (*n* = 75, 34.7%), followed by adolescents (*n* = 42, 19.5%). The majority of patients were males (*n* = 117, 54.2%). The most common etiologies of arrest were respiratory diseases (*n* = 66, 30.6%) and infections (*n* = 38, 17.6%). Asystole was the most predominant non-shockable cardiac rhythm (*n* = 148, 68.5%). Finally, most children received the first dose of epinephrine in the intermediate time category (*n* = 136, 63.0%), followed by early (*n* = 41, 18.9%), and late (*n* = 39, 18.1%).

**Table 1:** Demographics and resuscitation information.

**Variables**	**Non-shockable OHCA children who received epinephrine during resuscitation (*n* = 216)**	
	**No**.	**%**
**PATIENT CHARACTERISTICS**		
**Age Groups**		
Neonates	33	15.3
Infants	75	34.7
Toddlers	19	8.8
Preschool children	21	9.7
Schoolchildren	26	12.0
Adolescents	42	19.5
**Sex**		
Male	117	54.2
Female	99	45.8
**Etiologies of Arrest**		
Sudden infant death syndrome	31	14.3
Cardiovascular diseases	14	6.5
Neurological diseases	20	9.3
Respiratory diseases	66	30.6
Malignancy	16	7.4
Infections	38	17.6
Others	31	14.3
**PREHOSPITAL INFORMATION**		
Duration of prehospital CPR (mean ± SD, minutes) (7)^a^	11.3 ± 8.5	
The period from scene to hospital (mean ± SD, minutes) (8)^a^	13.5 ± 9.4	
Witness of arrest	148	68.5
Duration from time of collapse to EMS arrival (mean ± SD, minutes)	6.7 ± 5.2	
Duration of time from EMS arrival to epinephrine (mean ± SD, minutes)	7.5 ± 4.0	
**ED INFORMATION**		
**Initial Cardiac Rhythm**		
PEA	68	31.5
Asystole	148	68.5
Duration of in-hospital CPR	34.7 ± 23.5	
**ADMINISTRATION OF EPINEPHRINE**		
**First Epinephrine**		
Intravenous	196	90.7
Non-intravenous	20	9.3
**Time to Epinephrine^b^**		
Early (<15 min)	41	18.9
Intermediate (15–30 min)	136	63.0
Late (>30 min)	39	18.1
Number of epinephrine doses (times)	8.3 ± 6.2	

a *Number of patients with missing information*.

b *The duration from collapse (no pulse, no vital signs, and/or unconsciousness) to the first dose of epinephrine*.

### Primary Outcomes and Target Patient Identification

The primary outcomes are shown in Table 2. Among these children, 28 (13.0%) survived to discharge, and only 17 (7.9%) had good neurological outcomes. The number of patients who survived to ICU admission (target patients) was 73 (33.8%).

**Table 2:** Primary outcomes of patients.

**Outcomes**	**Non-shockable OHCA children who received epinephrine during resuscitation (*n* = 216)**	
	**No**.	**%**
Any ROSC	117	54.2
Sustained ROSC	86	39.8
Survived to ICU admission	73	33.8
Survived to discharge	28	13.0
Good neurological outcome	17	7.9

*ROSC, return of spontaneous circulation; ICU, intensive care unit*.

### Post-resuscitation Hemodynamics in Target Patients

The post-resuscitation hemodynamics (in the first hour of the post-resuscitation period) of the 73 targeted children are shown in Table 3. During this period, all patients were supported by ventilators. However, 17 (23.3%) of them still suffered hypoxia. Tachycardia (48.0%), sinus rhythm (68.5%), and hypertension (39.7%) were the most common cardiac presentations.

**Table 3:** Clinical features in the early post-resuscitation period.

**Variables of post-resuscitation hemodynamics**	**Time to epinephrine in children who survived to ICU admission (*n* = 73)**				***p***
	**Total**	**Early (*n* = 23)**	**Inter mediate (*n* = 41)**	**Late (*n* = 9)**	
	**No. (%)**	**No. (%)**	**No. (%)**	**No. (%)**	
**Oxygenation**					
Hypoxic	17 (23.3)	7 (30.4)	8 (19.5)	2 (22.2)	0.609
Non-hypoxic	56 (76.7)	16 (69.6)	33 (80.5)	7 (77.8)	
**Heart Rate^a^**					
Tachycardia	35 (48.0)	17 (73.9)	16 (39.0)	2 (22.2)	0.016
Normal	19 (26.0)	4 (17.4)	13 (31.7)	2 (22.2)	
Bradycardia	19 (26.0)	2 (8.7)	12 (29.3)	5 (55.6)	
**Heart Rhythm**					
Sinus	50 (68.5)	16 (69.6)	26 (63.4)	8 (88.9)	0.327
Non-sinus	23 (31.5)	7 (30.4)	15 (36.6)	1 (11.1)	
**Mean Blood Pressure**					
Hypertension	29 (39.7)	13 (56.5)	14 (34.1)	2 (22.2)	0.159
Normal	19 (26.0)	3 (13.0)	14 (34.1)	2 (22.2)	
Hypotension	25 (34.3)	7 (30.5)	13 (31.8)	5 (55.6)	
**Consciousness Level**					
GCS>7	13 (17.8)	6 (26.1)	5 (12.2)	2 (22.2)	0.697
GCS = 4–7	29 (39.7)	8 (34.8)	18 (43.9)	3 (33.3)	
GCS = 3	31 (42.5)	9 (39.1)	18 (43.9)	4 (44.5)	
**Body Temperature**					
Pyrexia	7 (9.6)	1 (4.3)	4 (9.8)	2 (22.2)	0.303
Non-pyrexia	66 (90.4)	22 (95.7)	37 (90.2)	7 (77.8)	
**Urine Output**					
>1 ml/kg/h	11 (15.1)	2 (8.7)	8 (19.5)	1 (11.1)	0.285
1–0.5 ml/kg/h	39 (53.4)	10 (43.5)	24 (58.5)	5 (55.6)	
<0.5 ml/kg/h	23 (31.5)	11 (47.8)	9 (22.0)	3 (33.3)	

a *Significant factor*.

*GCS, Glasgow Coma Score*.

### Secondary Outcomes—EE Influence on Hemodynamics

Among the target patients, 23 received EE. EE was significantly related to more predominant tachycardia (73.9%) than ME and LE (*p* < 0.05). However, EE was not significantly related to oxygenation, heart rhythm, blood pressure, level of consciousness, body temperature, or urine output in the early post-resuscitation period (Table 3).

### Most Powerful Effects of EE After Correction for Confounding Factors

After correction for the other confounding factors, multinomial logistic regression analysis demonstrated that EE was most closely related to tachycardia (OR: 7.41, 95% CI: 1.96–29.31), hypertension (OR: 6.03, 95% CI: 1.85–13.77), etiology of cardiovascular disease (OR: 3.21, 95% CI: 1.20–8.43), respiratory diseases (OR: 4.01, 95% CI: 1.21–36.44), duration from time of collapse to EMS arrival (OR: 0.92, 95% CI: 0.57–0.96), and duration of time from EMS arrival to epinephrine (OR: 0.87, 95%CI:0.67–0.98) (Table 4).

**Table 4:** Multinomial logistic regression analysis.

**Adjusted variables**	**Time to epinephrine**		
	**Early**	**Intermediate**	**Late**
	**OR (95% CI)**	**OR (95% CI)**	**OR (95% CI)**
**HEART RATE**			
Tachycardia^a^	7.41^a^ (1.96–29.31)	3.03 (0.75–11.01)	1
Normal^a^	5.90^a^ (1.33–32.41)	4.67 (0.85–25.75)	1
Bradycardia^b^	–	–	–
**HEART RHYTHM**			
Sinus	2.76 (0.80–17.43)	2.44 (0.74–11.90)	1
Non-sinus^b^	–	–	–
**MEAN BLOOD PRESSURE**			
Hypertension^a^	6.03^a^ (1.85–13.77)	3.17 (0.80–29.13)	1
Normal	2.80 (0.54–12.16)	3.43 (0.71–14.22)	1
Hypotension^b^	–	–	–
**CONSCIOUSNESS LEVEL**			
GCS>7	4.10 (0.90–12.54)	3.18 (0.71–19.31)	1
GCS = 4–7	2.51 (0.09–14.73)	2.33 (0.56–13.07)	1
GCS = 3^b^	–	–	–
**URINE OUTPUT**			
>1 ml/kg/h	2.41 (0.54–19.00)	3.46 (0.87–42.13)	1
1–0.5 ml/kg/h	1.20 (0.56–6.88)	2.33 (0.32–11.70)	1
<0.5 ml/kg/h^b^	–	–	–
**BODY TEMPERATURE**			
Non-pyrexia	2.54 (0.71–8.32)	1.54 (0.85–11.67)	1
Pyrexia^b^	–	–	–
**ETIOLOGIES OF ARREST**			
Sudden infant death syndrome	2.13 (0.73–14.29)	1.50 (0.37–16.80)	1
Cardiovascular diseases^a^	3.21^a^ (1.20–8.43)	2.19 (0.79– 10.02)	1
Neurological diseases	1.54 (0.38–11.07)	2.09 (0.61–13.93)	1
Respiratory diseases^a^	4.01^a^ (1.21–36.44)	1.98 (0.81–41.71)	1
Malignancy	1.24 (0.85–5.11)	4.20 (0.65–9.71)	1
Infections	2.76 (0.45–9.14)	3.34 (0.52–11.06)	1
Others^b^	–	–	–
**INITIAL CARDIAC RHYTHM**			
PEA	4.67 (0.93–4.77)	5.21 (0.96–5.33)	1
Asystole	–	–	–
Duration of prehospital CPR	0.98 (0.64–1.58)	0.96 (0.51–6.77)	0.95 (0.14–3.40)
Duration of in-hospital CPR	1.02 (0.89–4.01)	1.04 (0.93–5.60)	1.12 (0.77–2.96)
Duration from time of collapse to EMS arrival^a^	0.92 (0.57–0.96)^a^	0.95 (0.78–1.41)	0.94 (0.32–9.41)
Duration of time from EMS arrival to epinephrine^a^	0.87 (0.67–0.98)^a^	0.86 (0.55–5.29)	0.85 (0.83–6.11)

a *Significant impact factors*.

b *Reference group*.

### The Impact of EE on Primary Outcomes

**Figure 1:**
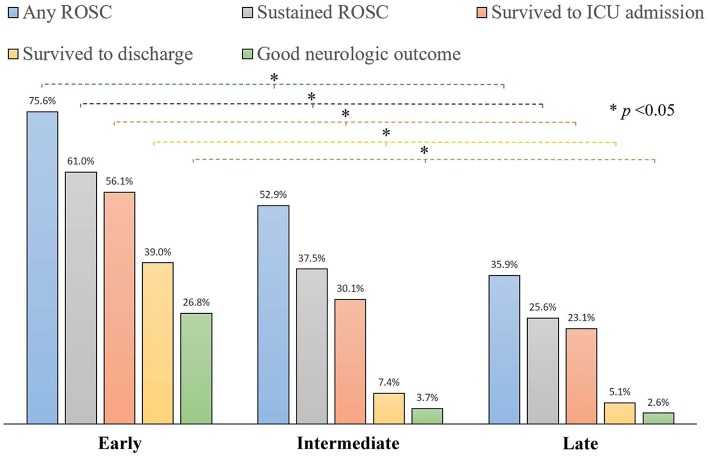
The associations between primary outcomes and different times to epinephrine in non-traumatic OHCA children (comparing EE, ME, and LE using the chi square test).

The associations between the primary outcomes and different times to epinephrine are shown in Figure 1. EE was associated with a greater chance of achieving any ROSC, sustained ROSC, survival to ICU admission, survival to discharge and good neurological outcomes than ME and LE (all *p* < 0.05).

## Discussion

Epinephrine is the first-line CPR medication and is only administered during the first medical contact (41). Thus, the “time to epinephrine” (from collapse to receiving the first dose of epinephrine) might differ significantly among OHCA patients. It is important to understand how patient outcome depends on the time to epinephrine. We suspect that EE might have more intense effects (more beneficial or harmful effects than ME or LE) on a well-established post-resuscitation hemodynamic status. However, this has never been demonstrated. In this study, we first analyzed the effects of EE on post-resuscitation hemodynamics in children with non-traumatic and non-shockable OHCA.

One important parameter influenced by EE is the post-resuscitation heart rate. Some studies have reported that the heart rate of OHCA patients was often increased and more variable during the ROSC period due to several factors, including hypoxia-related baroreceptor stimulation, ischemic/reperfusion response, inflammatory, and cytokine modulation and myocardial injuries (42–45). However, the association between EE and post-resuscitation tachycardia has not been well-addressed, especially in children with OHCA. Aside from other potential harmful effects, EE indeed triggered a greater heart rate in non-traumatic OHCA children.

The second key parameter triggered by EE was post-resuscitation hypertension. Blood pressure is directly associated with vasoconstriction (α-agonist effect) and cardiac output (β-agonist effect) (14, 46, 47). Therefore, we suspected that early administration might maximize the dual effects (α and β agonist effects) of epinephrine and then ROSC would be easier to establish. Increased blood pressure might not inevitably imply better cardiac output, as a higher blood pressure might just be the result of greater systemic vascular resistance (caused by epinephrine).

On the one hand, greater systemic vascular resistance might be detrimental to an end organ by limiting perfusion/cardiac output to that organ. On the other hand, it has been shown that EE provides benefit by increasing coronary artery perfusion pressure, and this might be associated with better outcomes (46, 48). Nevertheless, post-resuscitation oxygenation, rhythm, level of consciousness, body temperature, and urine output were not significantly associated with EE. This is compatible with the idea that EE might not directly improve end organ perfusion. However, more evidence is needed to prove this.

The most important finding was that survival rates and neurological outcomes were better in patients who received EE than in those who received ME or LE. This may be because EE rapidly triggers ROSC and thus immediately enhances blood flow to the brain.

In conclusion, EE not only helps to establish ROSC, but is also associated with cardiovascular effects in the early post-resuscitation period—inducing more tachycardia and hypertension, after correction for confounding factors. EE was associated with a higher survival rate and better neurological outcomes than ME and LE in children with non-traumatic OHCA.

## Limitations

There were four major limitations in this study. The first major limitation and a possible source of bias lay in the precise definition of “collapse”—as this could imply anything between full arrest and a low flow state. The second limitation lay in the determination of the true time points of collapse. As the time of collapse was retrospectively obtained from the medical/EMS records, we suspect that several factors (including differences in recording time, different definitions of collapse, and subjective effects of the witness) might influence the risk of bias. The third limitation could be the classification of initial cardiac rhythm. The rhythm seen first by first responders may not be the initial rhythm which caused the arrest. In addition, the initial shockable rhythms (VT/VF) might change to non-shockable (PEA/asystole) before EMS arrived (or even in the ambulance). The final limitation was that the influence of advanced cardiac life support (ALS) was not considered in this study. Because epinephrine was only administered by medical or EMS personnel, EE also indicated early ALS (including early airway management).

## Data Availability

The datasets for this manuscript are not publicly available because this is a medical chart review study. Requests to access the datasets should be directed to Yan-Ren Lin, h6213.lac@gmail.com.

## Ethics Statement

This study was carried out in accordance with the recommendations of STROBE statement. The protocol was approved by the institutional review board (IRB) of Changhua Christian Hospital. Since this is a retrospective chart-review study, and outputs were secondary deidentified data, informed consent were not needed under the IRB permission. Finally, the team members and data collector also followed the rules of the IRB.

1. Full name of the ethics committee: Institutional review board (IRB) of Changhua Christian Hospital.
2. Consent procedure used for human participants or for animal owners: retrospective chart-review review.
3. Any additional considerations of the study in cases where vulnerable populations were involved, for example minors, persons with disabilities or endangered animal species: Although we focused on OHCA children; however, this study only retrospectively obtained information from chart review (output data were also secondary deidentified). No additional considerations for vulnerable population under IRB permission.

## Author Contributions

W-LC, T-YC, C-JL, and Y-RL conceived the study. Y-RL and C-FC managed the data, including quality control. Y-RL, C-CH, and T-HL provided statistical advice on the study design and analyzed the data. C-JL, Y-RL, and C-CC chaired the data oversight committee. Y-RL and H-WH drafted the manuscript. H-YH and M-CY made a great contribution in 2nd and 3rd paper revisions. H-WH and W-LC assumed responsibility for the paper as a whole. All of the authors read and approved the final manuscript and contributed substantially to its revision.

### Conflict of Interest Statement

The authors declare that the research was conducted in the absence of any commercial or financial relationships that could be construed as a potential conflict of interest.
